# Serum anti-phospholipase A2 receptor (PLA2R) antibody detected at diagnosis as a predictor for clinical remission in patients with primary membranous nephropathy: a meta-analysis

**DOI:** 10.1186/s12882-019-1544-2

**Published:** 2019-09-18

**Authors:** Yufeng Liang, Jianxin Wan, Yongping Chen, Yangbin Pan

**Affiliations:** 1 Department of Nephrology, The Second Hospital of Longyan, Fujian, 364000 China; 2 Department of Nephrology, The First Affiliated Hospital of Fujian Medical University, Fuzhou, 35000 China

**Keywords:** Membranous nephropathy, M-type phospholipase A2 receptor antibody, Clinical remission, Meta-analysis

## Abstract

**Background:**

The diagnostic value of serum M-type phospholipase A2 receptor antibody (sPLA2R-ab) expression in patients with primary membranous nephropathy (PMN) has been established. However, the association between sPLA2R-ab and clinical remission remains uncertain.

**Methods:**

We systematically searched the literature for clinical trials regarding the correlation between sPLA2R-ab expression and clinical remission of PMN patients. Meta-analysis was performed to determine this association. Subgroup analysis, funnel plots, and sensitivity analysis were also performed to investigate heterogeneity or bias.

**Results:**

A total of 11 trials involving 824 patients were included. Patients with positive sPLA2R-ab had a poor clinical remission rate (RR = 0.76, 95%CI 0.68–0.86, *P* < 0.0001; *I*^*2*^ = 39%), a higher titer of sPLA2R-ab had a lower chance of clinical remission (RR = 0.72, 95%CI 0.59–0.87, *P* = 0.0006; *I*^*2*^ = 42%),and a higher risk of renal failure (RR = 4.85, 95% CI, 1.83–12.85, *P* = 0.002; *I*^2^ = 0%), without affecting relapse (RR = 0.97, 95% CI, 0.55–1.70; *P* = 0.92, *I*^2^ = 0%). Subgroup analysis by treatment strategies, assay methods, ethnicity, gender, renal function, the approach of ruling out SMN, and the ratio of patients with nephrotic-range proteinuria at baseline showed no significant association between these factors with the prognostic value of sPLA2R-ab for PMN patients. No significant publication bias was found.

**Conclusion:**

This meta-analysis adds to the evidence for current guidelines that sPLA2R-ab acts as not only a diagnostic marker but also a pivotal predictor for clinical remission. Therefore, sPLA2R-ab can be considered as a prognostic factor for stratifying PMN patients.

**Electronic supplementary material:**

The online version of this article (10.1186/s12882-019-1544-2) contains supplementary material, which is available to authorized users.

## Background

Primary membranous nephropathy (PMN) is a major cause of the nephrotic syndrome, which is characterized by subepithelial immune complex deposits with glomerular basement membrane thickening. The natural course of PMN is various, ranging from spontaneous remission to end-stage renal disease (ESRD). The disease process has been reported to be initiated by the binding of circulating autoantibodies to target podocyte antigens [1]. Proteinuria is the hallmark of PMN, whereas it is limited by the low sensitivity and specificity of diagnosis of early minimal lesions. Thus, searching for an efficacious biomarker for patients with PMN is warranted. Currently, serum M-type phospholipase A2 receptor antibody (sPLA2R-ab) is emerging as a predictive biomarker for early prognosis of PMN.

In 2009, Beck et al. [2] first found that PLA2R was abundantly expressed on human podocytes in 70% of patients with PMN. Recent evidence suggests that PLA2R autoantibodies play an important role in the diagnosis of PMN. Additionally, given the high specificity of sPLA2R-ab, the prognostic value of sPLA2R-ab has gained much interest of researchers. Many efforts have been devoted to assessing the association between sPLA2R-ab and clinical outcomes of PMN patients, including disease activity and remission. However, the results remain conflicting [1–6] regarding the prognosis of the value of sPLA2R-ab for PMN patients. These discrepancies may be attributed to the differences in ethnicity, immunosuppressive therapy, number of patients, and sPLA2R-ab testing methods [1, 2, 4, 5, 7–10]. In addition, the impact of sPLA2R-ab on clinical remission in PMN patients remains unclear. Currently, only one meta-analysis [11] has identified the impact of sPLA2R-ab on spontaneous remission in patients with PMN; however, it’s not exhaustive nor complete. To further address this issue, we performed a comprehensive meta-analysis to derive a more precise estimate of the prognostic value of the sPLA2R-ab among patients with PMN.

## Methods

### Search strategy

We intended to determine whether clinical remission of PMN was significantly associated with the titer of sPLA2R-ab. We searched PubMed, Web of Science, OVID, Cochrane, Chinese BioMedical Literature on disc (CBM), Chinese National Knowledge Infrastructure (CNKI), and WanFang databases. The following search terms were used: glomerulonephritis, membranous, M-type phospholipase A2 receptor antibody, cohort study, remission, and their synonyms and related terms. This meta-analysis was reported following the Preferred Reporting Items for Systematic reviews and Meta-Analyses (PRISMA) guidelines [12].

### Selection criteria

We collected all prospective cohort studies on the prognostic value of sPLA2R-ab in patients with PMN, which were published before 2019 Jan. The inclusion criteria were as follows: (1) cohort study, (2) patients were monitored for sPLA2R-ab levels with follow-up data, (3) patients were divided into groups with or without detectable sPLA2R-ab, (4) sPLA2R-ab testing methods included indirect immunofluorescence test (IIFT), the enzyme-linked immunosorbent assay (ELISA), or Western blotting (WB), (5) indications for immunosuppressive agents were determined by the physician based on the Kidney Disease Improving Global Outcomes (KDIGO) guidelines [6], which recommend immunosuppressive agents only in patients at a high risk for developing ESRD, (6) outcome measurements were complete remission (defined as proteinuria < 0.3 g/day) and partial remission (defined as proteinuria < 3.5 g/day but ≥0.3 g/day), the clinical remission included complete remission and partial remission. A relapse was defined as proteinuria > 3.5 g/d and an increase of > 50% compared with the lowest value during remission [10]. Renal failure (RF) was defined as a sustained increase of serum creatinine > 50% of baseline [4]. The exclusion criteria were as follows: (1) case reports, reviews, letters, editorials, or commentaries, (2) lack of a complete follow-up, (3) insufficient data to evaluate the prognosis, (4) overlapping subjects, (5) a small sample size (≤ 15), and (6) insufficient information.

### Data extraction and quality assessment

Two reviewers (YL and JW) independently extracted the data using a standardized data collection form. The following data were extracted: the last name of the first author, publication year, numbers of cases, sPLA2R-ab testing methods for sPLA2R-ab, follow-up duration, baseline laboratory parameters, and outcomes. Disagreements were resolved by a third reviewer (YP) who discussed with the two original reviewers. The methodological quality of studies was evaluated with the Newcastle-Ottawa Scale (NOS). The NOS is an 8-item instrument for quality assessment, and the grading standard was selection (0–4 points), comparability (0–2 points), and outcome (cohort studies, 0–3 points). These scores are listed in Table 1.

**Table 1:** Quality assessment (NOS scale, score)

Included studies	SELECTION				COMPARABILITY	EXPOSURE			NOS Score
	Is the case definition adequate?	Representativeness of the cases	Selection of controls	Definition of controls		Ascertainment of exposure	Same method of ascertainment for cases and controls	Non-response rate	
Hofstra JM 2012 [13]	1	1	1	1	1	1	1	1	8
Ruggenenti P 2015 [6]	1	1	1	0	1	1	1	1	7
Qin W 2011 [1]	1	1	1	0	1	1	1	1	7
Beck LH Jr. 2011 [9]	1	1	1	1	1	1	1	1	8
Kim YG 2015 [14]	1	1	1	1	1	1	1	0	7
Pourcine,F,2017 [7]	1	1	1	1	1	1	1	1	8
Oh YJ 2013 [15]	1	1	1	1	1	1	1	1	8
Timmermans SA 2015 [16]	1	1	0	1	1	1	1	1	7
Bech AP2014 [10]	1	1	1	1	1	0	1	1	7
Wei SY2016 [8]	1	1	1	1	1	1	1	1	8
Song EJ,2018 [17]	1	1	1	1	1	0	1	1	7

### Statistical analysis

Meta-analyses were performed by using Review Manager Version 5.1 (RevMan, Cochrane Collaboration) and STATA 12.0 statistical software (StataCorp, College Station, Tex). For dichotomous outcomes, relative risk (RR) with 95% confidence interval (CI) was the common measure of association across individual studies. Statistical heterogeneity among studies was evaluated with a Chi-square (*χ2*) test, and a value of *P* < 0.05 was considered statistically significant. Statistical heterogeneity was quantified with *I*^*2*^ tests and classified as low, moderate, and high for *I*^*2*^ values of 25, 50, and 75%, respectively. The meta-analysis was performed by using fixed-effects or random-effects methods according to the absence or presence of significant heterogeneity. A sensitivity analysis was conducted to assess the contribution of each study to the pooled RR. Publication bias was assessed using the Begg’s funnel plot.

## Results

### Study characteristics

**Fig. 1:**
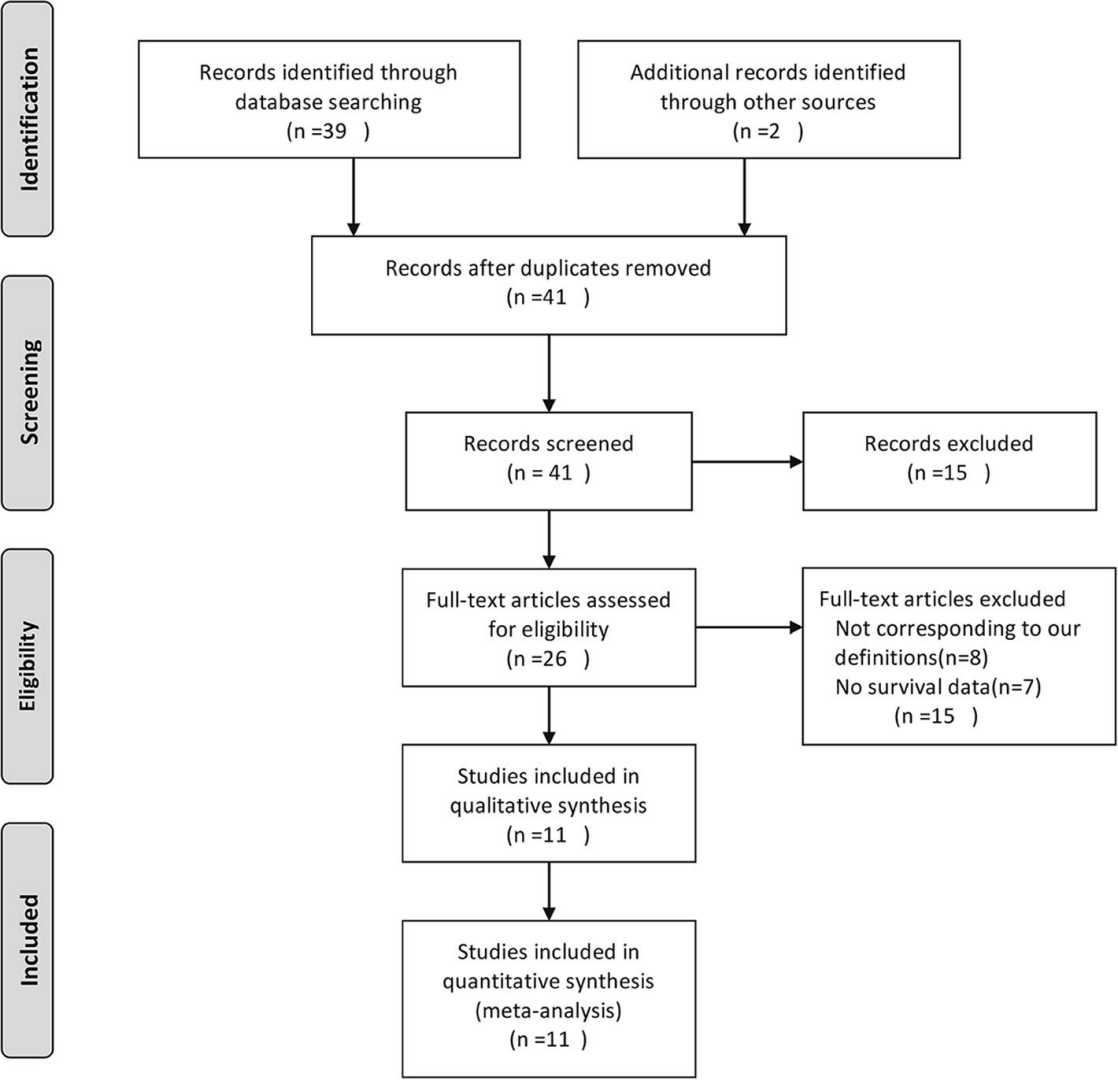
Flow diagram for identification, screening, and inclusion of papers for this meta-analysis

The literature selection process is presented in Fig. 1. Based on the search strategy, 41 relevant articles were retrieved. After reviewing the titles and full texts, 11 studies including 594 patients with positive sPLA2R-ab and 230 patients with negative sPLA2R-ab were finally included in our analysis. The characteristics and methodological quality of the included studies are shown in Table 2. The age at diagnosis of PMN by renal biopsy ranged from 34 [10] to 77 [4] years. The percentage of patients with positive sPLA2R-ab varied from 44.1% [14] to 89% [16]. The follow-up period ranged from 12 [1] to 168 [7] months.

**Table 2:** Baseline characteristics of the included studies

First author	Year	Race	Age	Sex (M/F)	Diagnosis	PRO (g/d)	CREA (mg/dl)	Fo (mo)	DD (mo)	IST n(n%)	TTR (mo)	Sample (n)	sPLA2R-ab(+) n (n%)	sPLA2R-ab (−) n (n%)	AM
Hofstra JM [13]	2012	No-As	51.6 ± 16.0	96/26	RB	10.2 (3.6–37.9)	95 (51–302)	54	NA	NA	NA	109	79 (72.5%)	28 (25.7%)	ELISA /IIFT
Ruggenenti P [6]	2015	No-As	55.7 ± 15.4	100/32	RB	9.1 (5.8–12.7)	1.21 (1.00–1.73)	144	25.8 (11.0–70.3)	49 (37.7)	NA	101	81 (80.2%)	20 (19.8%)	ELISA
Qin W [1]	2011	As	47.2 ± 15.4	16/44	RB	5.42 (4.39–7.61)	0.85 ± 0.5	> 12	3.0 (1.25–8.25)	NA	12 (6.5–22)	60	49 (81.7%)	11 (18.3%)	WB
Beck LH Jr [9]	2011	No-As	48.2 ± 11.1	5/30	RB	10.8 (5.7–26.5)	1.4 (1.00–1.80)	24	10 (6–17)	NA	NA	35	25 (71.4%)	10 (28.6%)	WB
Timmermans SA [16]	2015	No-As	52.4 ± 14.0	44/29	RB	6.7 (4.0–9.7)	0.97 (0.89–1.23)	34.8	NA	26 (35.6%)	NA	73	65 (89.0%)	8 (11.0%)	ELISA
Oh YJ [15]	2013	As	55.6 ± 13.9	56/77	RB	6.07 (3.17–9.86)	0.92 ± 0.35	30	NA	NA	2.0 (1.0–4.0)	77	56 (72.7%)	21 (27.3%)	WB
Kim YG [14]	2015	As	50.7 ± 15.0	15/19	RB	6.55 ± 3.61	0.76 ± 0.14	12	NA	NA	NA	93	41 (44.1%)	52 (55.9%)	ELISA
Bech AP [10]	2014	No-As	55(34–7)	10/37	RB	10.1 (3.2–25.2)	1.60 (0.98–3.37)	60	5 (1–26)	4 (8.3%)	NA	48	34 (70.8%)	14 (29.2%)	ELISA
Wei SY [8]	2016	As	48.2 ± 12.7	72/41	RB	10.78 ± 6.81	NA	≤20	NA	NA	NA	93	55 (59.1%)	38 (40.9%)	WB
Pourcine F [7]	2017	No-As	54(40.5–65.1)	32/53	RB	7.1 (3.5–10.8)	NA	168	NA	NA	NA	85	46 (54.1%)	22 (25.9%)	ELISA
Song EJ [17]	2018	As	55.35 ± 13.33	29/19	RB	6.20 ± 3.66	0.81 ± 0.33	65	NA	NA	NA	48	25 (52.1%)	23 (47.9%)	ELISA

*JASN* J Am Soc Nephrol, *CJSN* Clin J Am Soc Nephrol, *mo* month, *No-As* none Asian, *As* Asian, *M* male, *F* female, *PRO* proteinuria, *CREA* serum creatinine, *Fo* follow up, *TTR* time to remission, *RB* renal-biopsy, *DD* disease duration, *AM* assay method, *WB* Western blotting, *ELISA* enzyme-linked immunosorbent assay, *IIFT* indirect immunofluorescence staining, *IST* immunosuppressive treatment

### Effect of sPLA2R-ab on the rate of clinical remission

**Fig. 2:**
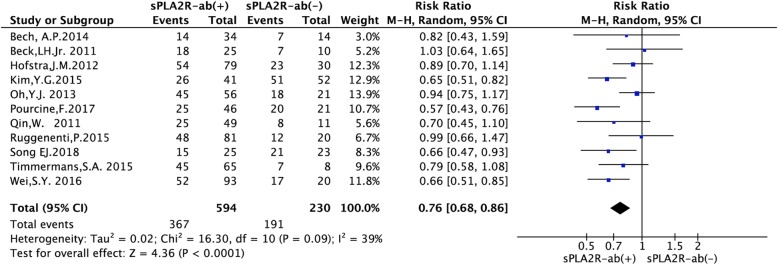
Forest plot for the correlation between sPLA2R-ab and the rate of clinical remission in patients with IMN

As shown in Fig. 2, we compared the positive and negative categories from 11 studies to summary the RR [1, 6–10, 13–17]. Among 594 sPLA2R-ab positive patients and 230 sPLA2R-ab negative patients, the rate of clinical remission was 61.78% and 83.04%, respectively (RR = 0.76, 95% CI, 0.68–0.86; *P* < 0.0001). Low heterogeneity was noted (*P* = 0.09, *I*^*2*^ = 39%). Therefore, a random-effects model was selected. We observed a significant decrease in the rate of clinical remission among patients with positive sPLA2R-ab.

### Effect of sPLA2R-ab titer by ELISA on the rate of clinical remission

**Fig. 3:**
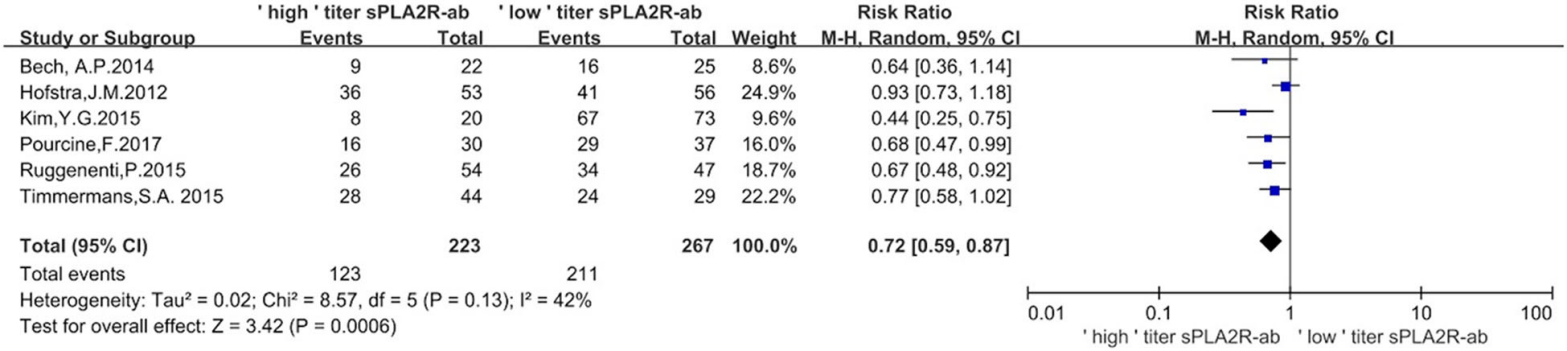
Forest plot for the correlation between ‘low’ or ‘high’ sPLA2R-ab titer by ELISA and the rate of clinical remission

To evaluate the association of the titer with the rate of clinical remission, patients were divided into ‘low’ or ‘high’ titer based on the original research, which had clarified the value of sPLA2R-ab titer from ELISA. The ‘low’ titer sPLA2R-ab group was defined as antibody levels in the lowest tertile and seronegative patients, whereas the ‘high’ titer sPLA2R-ab group was defined as antibody levels in the middle and highest tertile. As shown in Fig. 3, the rate of clinical remission was 55.16% and 79.03%, respectively (RR = 0.72, 95% CI, 0.59–0.87; *P* = 0.0006). Low heterogeneity was noted (*P* = 0.13, *I*^2^ = 42%). Therefore, a random-effects model was selected. The clinical remission rate was higher in patients with ‘low’ titer of sPLA2R-ab (Fig. 3).

### Subgroup analysis

**Fig. 4:**
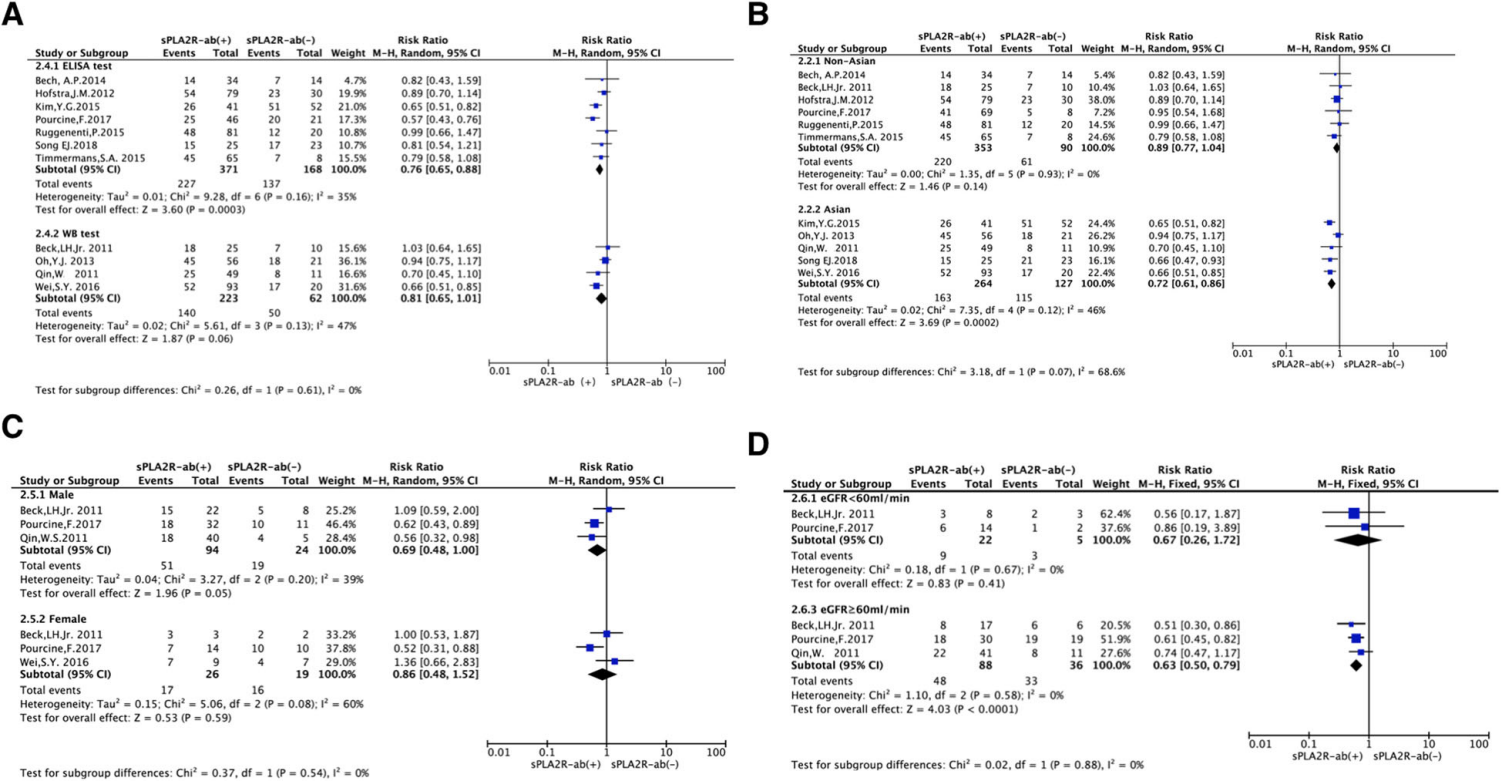
Forest plot for the correlation between sPLA2R-ab and the rate of clinical remission based on assay methods (**a**), ethnicity (**b**), gender (**c**), and renal function (**d**)

To further assess the impact of sPLA2R-ab on clinical remission, we performed subgroup analysis of the pooled results according to assay methods, ethnicity, gender, and baseline renal function (Fig. 4), whether detection of gPLA_2_R and/or its mainly subclass IgG4 as powerful approach for ruling out secondary forms of MN (SMN,Additional file 1: Figure S1A) and whether all patients with nephrotic-range proteinuria at baseline (Additional file 1: Figure S1B). Subgroup analysis demonstrated that the heterogeneity wasn’t eliminated. The pooled results of including studies demonstrated that sPLA2R-ab positive patient had lower clinical remission rate in the ELISA assay subgroup (RR = 0.82, 95% CI, 0.72–0.93; *P* = 0.002, Fig. 4a). Similarly, sPLA2R-ab positive patient had lower clinical remission rate in the Asian subgroup (RR = 0.36; 95% CI, 0.21–0.62; *P* = 0.0002, Fig. 4b), as well as patients with baseline renal function above 60 ml/min (RR = 0.63, 95% CI, 0.50–0.79; *P* < 0.0001, Fig. 4c).

### Effect of sPLA2R-ab on the rate of spontaneous remission and drug-induced remission

**Fig. 5:**
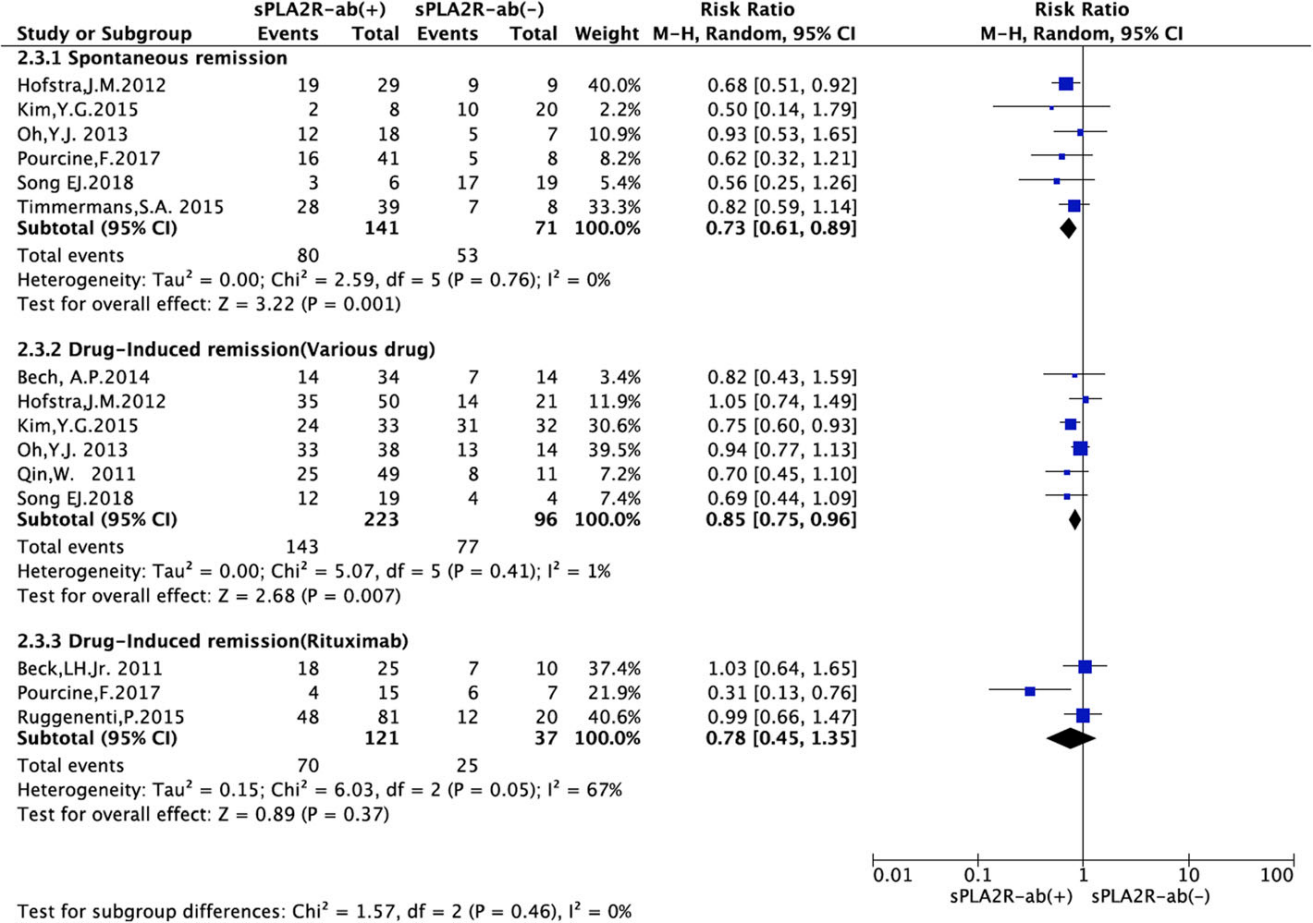
Forest plot for the correlation between sPLA2R-ab and the rate of clinical remission based on different treatment strategy

Spontaneous remission tended to occur slightly more often in the sPLA2R-ab negative patients (RR = 0.73, 95% CI, 0.61–0.89; *P* = 0.001). A total of 319 patients received immunosuppressive agents (e.g. adrenocorticotrophic hormone, cyclophosphamide, and mycophenolate mofetil). In the immunosuppressive agent subgroup, remission developed after immunosuppressive treatment and tended to occur more often in the sPLA2R-ab negative patients (RR = 0.85, 95% CI, 0.75–0.96; *P* = 0.007, Fig. 5).

### Effect of sPLA2R-ab on the rate of renal failure

**Fig. 6:**
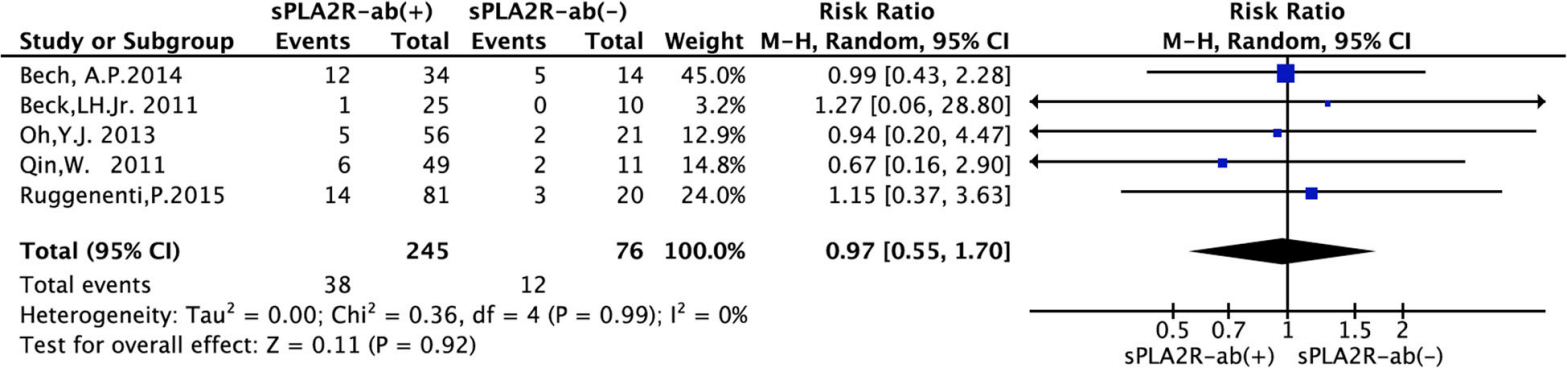
Forest plot for the correlation between sPLA2R-ab and the rate of renal failure in patients with IMN

Three studies with 224 patients assessed the association of sPLA2R-ab and the rate of renal failure in PMN. The results of meta-analysis for renal failure showed that patients with positive sPLA2R-ab had a significantly higher rate of renal failure compared to patients with negative sPLA2R-ab (RR = 4.85, 95% CI, 1.83–12.85; *P* = 0.002), indicating significant homogeneous across these studies (*P* = 0.74, *I*^2^ = 0%, Fig. 6).

### Effect of sPLA2R-ab on the rate of relapse

**Fig. 7:**
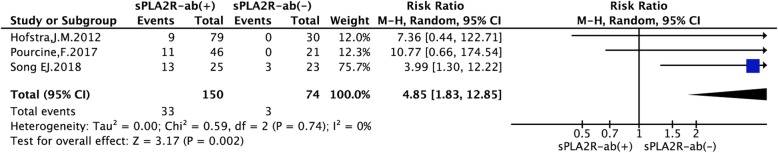
Forest plot for the correlation between sPLA2R-ab and the rate of relapse in patients with IMN

Five studies with 321 patients assessed the association of sPLA2R-ab with relapse in PMN. The results of meta-analysis for relapse showed that patients with positive sPLA2R-ab at diagnosis had a similar rate of relapse compared to patients with negative sPLA2R-ab (RR = 0.97, 95% CI, 0.55–1.70; *P* = 0.92) without significant heterogeneity (*P* = 0.99, *I*^2^ = 0%, Fig. 7).

### Sensitivity analysis and publication bias

A sensitivity analysis for the rate of remission was performed to assess the effect of study quality on the stability of this meta-analysis, and the results were consistent with those of the main meta-analysis, suggesting reliable findings of this meta-analysis (Additional file 2, Figure S2). Publication bias was assessed by Begg’s funnel plot analysis (*P* = 0.876), and Egger’s linear regression test was used to verify the accuracy of Begg’s funnel plot (*P* = 0.896). No evidence of significant publication bias was found, as shown in Additional file 2: Figure S2).

## Discussion

The prognostic value of the sPLA2R-ab expression for PMN patients has been well established; however, the results remain controversial. This may be attributed to treatment strategies, detected method, ethnicity, baseline renal function. Herein, for the first time, this meta-analysis examined the prognostic value of the sPLA2R-ab expression in PMN patients. We demonstrated that sPLA2R-ab at diagnosis could be considered as a prognostic biomarker for stratifying PMN patients.

PLA2R is the major target autoantigen in PMN [2], which plays an important role in the pathogenesis and clinical progression. Detection of circulating autoantibodies binding to PLA2R (detected by WB [1, 2, 4], or ELISA [16, 18–20]) is an important clue to the diagnosis of PMN. In addition, concentrations of sPLA2R-abs correlate with disease activity of PMN. Recently, a number of studies [1, 4–7, 12–17] have assessed the association between sPLA2R-ab and clinical outcome of PMN, including loss of renal function, clinical remission, time to remission. However, these studies were performed on limited sample size. Therefore, to evaluate the impact of PLA2R-Abs expression on the clinical outcome, we integrated high-quality studies and performed this meta-analysis. The results demonstrated that compared with PMN patients with positive sPLA2R-ab, PMN patients with negative sPLA2R-ab were associated with the rate of clinical remission regardless of the prescription of conservative treatment or immunosuppressive agent. Additionally, we found negative sPLA2R-ab patients were correlated with a lower rate of renal failure.

In subgroup analysis, measurement of sPLA2R-ab by ELISA assay had a more significant prognostic value than that by IFFT assay, indicating a better specificity in predicting clinical remission in patients with IMN. In patients detected sPLA2R-ab by ELISA assay, we noticed that patients with a higher titer of sPLA2R-ab at the initiation of treatment had a lower probability of the clinical remission. An elevated sPLA2R-ab in Asian group had a more significant prognostic implication than in the non-Asian group, suggesting a better specificity of positive sPLA2R-ab in predicting poor prognosis in Asian patients with IMN. We demonstrated sPLA2R-ab in the group CKD stage> 3 before treatment had a more significant prognostic significance, indicating that sPLA2R-ab in a worse renal function is more specific to predict a poor prognosis in patients with IMN. Despite the limited number of the eligible studies in this meta-analysis, the pooled results showed that an elevated sPLA2R-ab is associated with a poor prognosis in patients with IMN.

The heterogeneity between studies was relatively small. One of the possible explanations could be attributed to the assay methods, ethnicity, gender, baseline renal function, the approach of ruling out SMN, and the ratio of patients with nephrotic-range proteinuria at baseline. In subgroup analysis, there was no evidence showing that the prognostic value of sPLA2R-ab was affected by factors included in the analysis. Additionally, funnel plot and sensitive analysis in our meta-analysis indicated that the pooled results were relatively conclusive.

Having shown the association between the sPLA2R-ab and the clinical remission and renal failure, the correlation between sPLA2R-ab and treatment relapse remains unclear. However, previous studies [6, 9] have indicated that dynamic monitoring sPLA2R-ab in patients with PMN during follow-up correlate with long-term outcome, partial or complete depletion of sPLA2R-ab preceded renal remission. On the other hand, expression of sPLA2R-ab at the end of immunosuppressive treatment predicts the occurrence of relapse, indicating that sPLA2R-ab is associated with clinical outcome. Furthermore, sPLA2R-ab may play a pathogenic role in PMN, it might be explained by deposit reconstructive and restoration of the glomerular capillary wall [19]. More well-designed studies, especially randomized controlled trials, should focus on the elimination of sPLA2R-ab in order to improve renoprotection.

Our study had some limitations. First, only 824 patients were included in these studies; therefore, a large-scale population-based study was warranted. Second, subgroup analysis according to alternative target antigens, such as THAS7D, was not performed due to the limited number of publications [21–26]. Third, only three original publications reported sPLA2R-ab in PMN patients, which increased the sensitivity for the diagnosis. Forth, this meta-analysis was conducted in the absence of a registered protocol, without other languages included in the study. Fifth, the literature search was not conducted among conference abstract databases and relevant society websites, and it may introduce bias. However, we did not consider the levels of PLA2R antigens in glomerular deposits and their clinical significance (Additional file 3: Table S1). Compared with kidney biopsies, sPLA2R-ab detection is considered as noninvasive and more readily accepted by patients. Finally, a limited number of studies have explored the dynamic monitoring sPLA2R-ab relationship with long-term outcome. Further research is required to assess the association and provide evidence to eliminate sPLA2R-ab and renoprotection.

## Conclusions

In conclusion, this meta-analysis adds to the evidence for current guidelines that sPLA2R-ab acts as not only a diagnostic marker but also a pivotal predictor for clinical remission. Therefore, sPLA2R-ab can be considered as a contributing prognosis factor for stratifying PMN patients.

## Additional files

Additional file 1: **Figure S1.** Forest plot for the correlation between sPLA2R-ab and the rate of clinical remission based on the approach for ruling out SMN (A), whether all patients with nephrotic-range proteinuria at baseline (B). (TIF 209 kb)

Additional file 2: **Figure S2.** Sensitivity analysis and Funnel plot analysis of potential publication bias (Begg’s test). (DOCX 265 kb)

Additional file 3: **Table S1.** only three studies had reported sPLA2R in patients with IMN. (DOCX 17 kb)

## Data Availability

All data that support the conclusions of this manuscript are included within the article.

## Abbreviations

CI: Confidence interval
PMN: Primary membranous nephropathy
RR: Relative risk
sPLA2R-ab: Serum phospholipase A2 receptor antibody
