# Supplementary figures and images for: Serum anti-phospholipase A2 receptor (PLA2R) antibody detected at diagnosis as a predictor for clinical remission in patients with primary membranous nephropathy: a meta-analysis
Source: BMC Nephrol. 2019 Sep 18;20:360. doi: 10.1186/s12882-019-1544-2 (PMC6749720; doi:10.1186/s12882-019-1544-2)

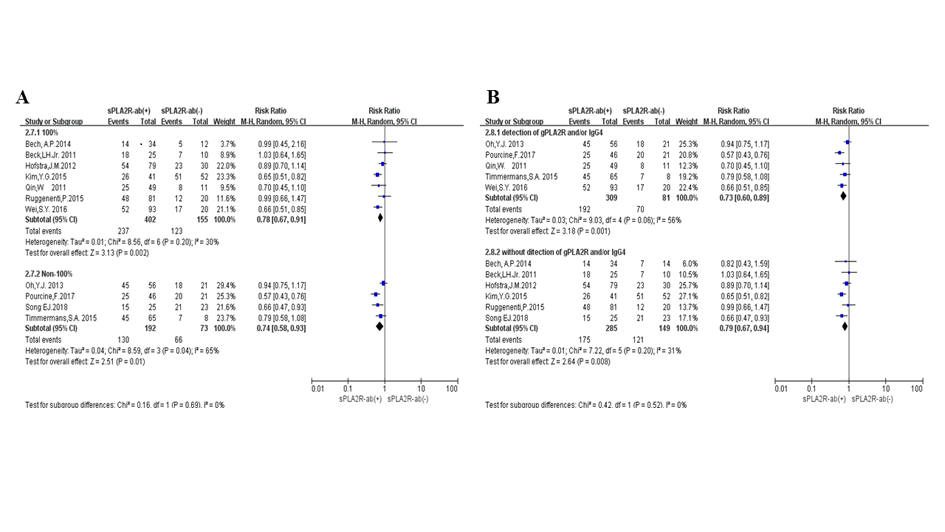

Supplement: Supplementary file 1 — Figure S1. Forest plot for the correlation between sPLA2R-ab and the rate of clinical remission based on the approach for ruling out SMN (A), whether all patients with nephrotic-range proteinuria at baseline (B). (TIF 209 kb) [file 12882_2019_1544_MOESM1_ESM.tif]
