# Supplementary material for: Serum anti-phospholipase A2 receptor (PLA2R) antibody detected at diagnosis as a predictor for clinical remission in patients with primary membranous nephropathy: a meta-analysis
Source: BMC Nephrol. 2019 Sep 18;20:360. doi: 10.1186/s12882-019-1544-2 (PMC6749720; doi:10.1186/s12882-019-1544-2)
Supplement: Supplementary file 2 — Figure S2. Sensitivity analysis and Funnel plot analysis of potential publication bias (Begg’s test). (DOCX 265 kb) [file 12882_2019_1544_MOESM2_ESM.docx]

0.65

0.77

0.68

0.87

0.90

Wei,S.Y (2016)

Bech, A.P. (2014)

Beck,LH.Jr. (2011)

Hofstra,J.M (2012)

kIM,Y.G (2015)

Oh,Y.J (2013)

pourcine,F (2017)

Qin,W (2011)

Ruggenenti,P (2015)

song,EJ. (2018)

Timmermans,S.A (2015)

Lower CI Limit

Estimate

Upper CI Limit

Meta-analysis estimates, given named study is omitted
