# Supplementary material for: Serum anti-phospholipase A2 receptor (PLA2R) antibody detected at diagnosis as a predictor for clinical remission in patients with primary membranous nephropathy: a meta-analysis
Source: BMC Nephrol. 2019 Sep 18;20:360. doi: 10.1186/s12882-019-1544-2 (PMC6749720; doi:10.1186/s12882-019-1544-2)
Supplement: Supplementary file 3 — Table S1. only three studies had reported sPLA2R in patients with IMN. (DOCX 17 kb) [file 12882_2019_1544_MOESM3_ESM.docx]

| **Table S1 only three studies had reported sPLA2R in patients with IMN** | | | | | |
| --- | --- | --- | --- | --- | --- |
|  | PLA2R subclass | Positivity (%) | Clinical correlation (s) | Correlation with sPLA2R ab | Comments |
| Qin W | IgG4 | 49(60) | Not mentioned | Not correlate with the titre of sPLA2R | IgG4 deposition in patients with IMN whose sPLA2R antibodies were positive |
| Timmermans SA | Not mentioned | | | | Increase the sensitivity for the diagnosis |
| Wei SY | IgG4 | 97(113) | Without correlation | Both positive in IMN | No correlation between the intensity of PLA2R staining and prognosis |
| Abbreviations: sPLA2R: glomerular PLA2R expression; IMN: idiopathic membranous nephropathy; sPLA2R ab: anti-phospholipase A2 receptor; | | | | | |
